# Changes of regional brain activity in frontal areas associated with cognitive impairment in obstructive sleep apnea-hypopnea syndrome patients: a resting-state fMRI study

**DOI:** 10.3389/fnins.2025.1587180

**Published:** 2025-06-17

**Authors:** Mei Jing, Min Guo, Xinrui Wang, Peng Zhang, Chenchen Gu, Nan Zhang, Zhiqiang Ding, Kelei Su

**Affiliations:** ^1^Department of Radiology, Affiliated Hospital of Integrated Traditional Chinese and Western Medicine, Nanjing University of Chinese Medicine, Nanjing, Jiangsu, China; ^2^Jiangsu Province Academy of Traditional Chinese Medicine, Nanjing, Jiangsu, China; ^3^Department of Otolaryngology, Affiliated Hospital of Integrated Traditional Chinese and Western Medicine, Nanjing University of Chinese Medicine, Nanjing, Jiangsu, China; ^4^Department of Respiratory and Critical Care Medicine, Affiliated Hospital of Integrated Traditional Chinese and Western Medicine, Nanjing University of Chinese Medicine, Nanjing, Jiangsu, China

**Keywords:** obstructive sleep apnea-hypopnea syndrome, resting-state functional magnetic resonance imaging, polysomnography, Montreal cognitive assessment, cognitive impairment

## Abstract

**Introduction:**

Obstructive sleep apnea-hypopnea syndrome (OSAHS) can lead to cognitive impairment, however, its central neural mechanism is still unclear.

**Methods:**

Resting-state functional magnetic resonance imaging (rs-fMRI) data were collected from 31 severe OSAHS patients and 28 healthy controls (HCs). Both regional homogeneity (ReHo) and fractional amplitude of low-frequency fluctuation (fALFF) measures were calculated and compared between groups. Moreover, the correlations between abnormal regions and Montreal Cognitive Assessment (MoCA) scores were evaluated using Pearson correlation analysis. Finally, ROC analysis was performed to evaluate the values of abnormal brain regions for distinguishing OSAHS patients from HCs.

**Results:**

OSAHS patients had decreased MoCA scores when compared with HCs. In comparison with HCs, increased ReHo values were found in the left and right rolandic operculum of OSAHS patients. In addition, decreased fALFF values were identified in the right superior frontal gyrus, right postcentral gyrus, left angular gyrus while increased fALFF values were identified in the left thalamus, right thalamus and right putamen of OSAHS patients. Positive relationships were found between fALFF values of the right superior frontal gyrus and MoCA scores in the patient group. The results of ROC analysis showed that the combined model of (ReHo and fALFF values of all abnormal brain regions) could effectively distinguish OSAHS from HCs.

**Conclusion:**

Severe OSAHS patients showed decreased brain activities, which were associated with the decreased cognition of patients. In addition, abnormal brain regions could help distinguishing OSAHS patients from HCs. These findings provided new insights about the potential pathogenesis of cognitive impairment caused by OSAHS from the perspective of changes in brain activity.

## 1 Introduction

Obstructive sleep apnea-hypopnea syndrome (OSAHS) is a common sleep apnea disorder, which is characterized by repeated upper respiratory tract during sleep, obstruction and/or collapse, resulting in respiratory pauses or inadequate ventilation (Lee and Sundar, 2021). About 24% of adult males and 9% of adult females experience changes in their physical and mental health due to OSAHS (Peppard et al., 2013). OSAHS is closely related to increased incidence rate of cardiovascular and cerebrovascular diseases and metabolic diseases (Redline et al., 2023; Gottesman et al., 2024; Zhang et al., 2021). Like diabetes, hypertension and hyperlipidemia, OSAHS is an important chronic disease that affects human health (Read et al., 2023). Compared to the general population, the total risk of death for OSAHS individuals is approximately 5.3 times higher (Marshall et al., 2014). Multiple factors combine to cause airway obstruction, leading to respiratory pauses or low ventilation (Lee and Sundar, 2021). Previous studies have confirmed that the occurrence and development of OSAHS is a multifactorial process, which is related to various systems in the body, including the formation of reactive oxygen species, abnormal activation of metabolism and inflammatory responses (Zhang et al., 2020; Lu et al., 2018; Lavie and Lavie, 2009). Common risk factors for OSAHS include obesity, advanced age, male and female postmenopausal status, race, and craniofacial abnormalities (Ge et al., 2024). OSAHS itself can also cause obesity due to insufficient sleep at night, metabolic interruption, and other factors (Feng et al., 2024). Epidemiological study has found that a 10% increase in body mass index (BMI) is associated with a 32% increase in the Sleep Apnea Hypoventilation Index (AHI), indicating a correlation between high BMI and the incidence of OSAHS (Grewal and Joshi, 2019). The occurrence of OSAHS symptoms varies greatly among individuals, and patients may present with one or more symptoms, or may have no symptoms at all (Maspero et al., 2015). If the condition does not improve for a long time, it will also increase the risk of cardiovascular and cerebrovascular diseases (Wang et al., 2023; Liu et al., 2020).

The main symptoms of patients are respiratory pauses witnessed by others, sleep disturbances and poor quality, daytime sleepiness, followed by memory decline, and in some cases, cognitive decline, behavioral abnormalities, other complications, and systemic target organ damage (Platon et al., 2023). Repeated occurrence of sleep apnea and/or hypopnea, leading to nocturnal hypoxemia, fragmented sleep, excessive daytime sleepiness, impaired behavior and cognitive function, emotional disorders, and other systemic damages (Kinugawa, 2021; Pan et al., 2024). These factors collectively participate in the process of cognitive decline. Epidemiological study showed that 5–15% of patients with mild cognitive impairment developed dementia each year, accompanied by pathological changes that were difficult to reverse, resulting in the loss of independent living and self-care abilities in the later stages, which imposed a huge economic burden on individuals and society (Dunne et al., 2021). However, previous research both domestically and internationally is mainly focused on the impact of OSAHS on cardiovascular and cerebrovascular risks, metabolic syndrome, and respiratory diseases, with less attention paid to cognitive impairment. Research has shown that intermittent hypoxia caused by sleep apnea at night is a special high-frequency hypoxia pattern, with severe hypoxia and large fluctuations in blood oxygen levels, which can lead to an imbalance of the antioxidant defense system, functional damage to neurons resulting in changes in brain metabolites, and can also promote the occurrence of cerebral small vessel disease, leading to lacunar infarction, white matter abnormalities and gray matter loss, disruption of brain connections, and affecting cognitive function (Polsek et al., 2018; Zinchuk et al., 2017).

Narrowing and occlusion of the respiratory tract can lead to increased brain plasticity and vascular responses in patients during periods of nocturnal respiratory pauses and low breathing episodes (Toth et al., 2012). The pathways activated by the central nervous system may cause functional changes in the patient’s brain, resulting in impaired brain activity and emotional and cognitive changes, such as depression, attention disorders, thinking and emotional disorders, executive and verbal memory disorders, etc. (Baima et al., 2020; Castronovo et al., 2009; Luo et al., 2015). With the development of technology, functional magnetic resonance imaging (fMRI), as a non-invasive detection method, is gradually being applied to explore the potential neuroimaging mechanisms of cognitive impairment by evaluating the changes of brain activity (Chandra et al., 2019; Yue et al., 2023). Based on the blood oxygen level dependent (BOLD) effect, fMRI is mainly to detect changes in brain signals, reflecting changes in the ratio of oxygenated hemoglobin/deoxygenated hemoglobin in the brain (D'esposito et al., 2003). The changes in blood oxygen levels are actually caused by changes in local cerebral blood flow, which are often closely related to local brain activity (Ekstrom, 2010). The characteristic of OSAHS is long-term intermittent hypoxia, and the brain is an extremely sensitive organ to hypoxia (Burtscher et al., 2021). Therefore, the method of fMRI can be used to evaluate the changes of brain activity in OSAHS patients, especially those with cognitive impairment (Li et al., 2015). The use of resting-state fMRI (rs-fMRI) for assessing cognitive impairment in OSAHS patients has the following advantages: (1) non-invasive: rs-fMRI is a non-invasive method, especially suitable for OSAHS patients who are usually in poor physical condition and unable to bear the burden of invasive examinations; (2) objectivity: compared with traditional cognitive function assessment scales, rs-fMRI can provide more objective and accurate assessment results, which helps to more accurately understand the functional status of the brain in OSAHS patients; (3) high spatial resolution: rs-fMRI can clearly display the anatomical structure and functional activity areas of the brain, accurately locate brain regions related to cognitive function, and observe the activity changes of these brain regions to gain a deeper understanding of the neural mechanisms underlying cognitive dysfunction in OSAHS patients.

In the resting state, the brain also exhibits spontaneous, organized, and continuous activity, which is closely related to cognitive function. When the brain engages in cognitive activity, local neuronal activity increases, which can cause changes in local blood flow and oxygen levels, leading to changes in magnetic resonance signal intensity. The rs-fMRI utilizes this BOLD effect to reflect the activity of the brain by detecting spontaneous fluctuations in blood oxygen levels at rest. The fractional amplitude of low-frequency fluctuation (fALFF) suggests spontaneous activity of brain neurons by directly observing the amplitude of BOLD signal changes relative to baseline, and an increase in fALFF value indicates an increase in the intensity of spontaneous brain activity. The regional homogeneity (ReHo) reflects the temporal synchronicity of neural activity within a local brain region by calculating the Kendall coefficients of a given voxel in a time series with multiple other voxels. An increase or decrease in ReHo values in a local brain region indicates a change in the consistency of neuronal activity, suggesting the possibility of functional abnormalities in that region and reflecting the strength of spontaneous brain activity. Patients with cognitive impairment may experience abnormal spontaneous brain activity. For patients with mild cognitive impairment, there may be an increase or decrease in ReHo values, fALFF values, etc., which are correlated with the results of neuropsychological tests.

At present, there are relatively few studies on the changes of brain activity in OSAHS patients, and the results have been inconsistent. Based on the previous research results, the aim of this study was to extend our understanding of brain dysfunction of OSAHS patients. In this study, we hypothesized that OSAHS could impair cognitive function through brain activity, and based on rs-fMRI data, ReHo and fALFF measures might exhibit significant differences between severe OSAHS patients and healthy controls (HCs), which could identify brain regions with functional changes related to cognitive impairment of OSAHS patients.

## 2 Materials and methods

### 2.1 Participants

This study was approved by the Ethics Committee of Affiliated Hospital of Integrated Traditional Chinese and Western Medicine, Nanjing University of Chinese Medicine (NO. 2023-LWKY073) and was conducted in accordance with the Helsinki Declaration of the World Medical Association. In addition, written consents were collected form all participants participating in this study. A total of 31 untreated severe OSAHS patients diagnosed by polysomnography (PSG) and 28 HCs were included in this study. The diagnosis of severe OSAHS were according to the American Academy of Sleep Medicine (AASM) guidelines (Kapur et al., 2017) and the diagnostic criteria were as follows: (1) patients typically experience daytime sleepiness, severe snoring during sleep, and repeated episodes of respiratory pauses; (2) PSG monitored AHI ≥ 30times/h and nighttime minimum SaO_2_ (%) ≤ 80% during the 7-h sleep process; (3) could not be attributed to other sleep disorders, such as simple snoring. All HCs had normal physical examination results without clinical symptoms of OSAHS and intracranial disease as screened by professional physicians. In this study, all participants underwent neuropsychological assessment with the Montreal Cognitive Assessment (MoCA) administered and scored by an experienced doctor based on published procedures.

The inclusion criteria were as follow: all participants were right-handed Han Chinese, between 20 and 60 years of age and had no less than 9 years of education. The exclusion criteria were as follow: (1) sleep disorder other than OSAHS; (2) history of treatment with drugs or surgery for OSAHS; (3) history of brain injury or positive nervous system examination; (4) history of mental or neurological disorders; (5) uncontrolled or serious physical disease, such as cardiovascular and respiratory diseases, diabetes mellitus or thyroid disease and other related diseases; (6) history of drug abuse, alcoholism, or psychotropic drug use; (7) MRI contraindications such as claustrophobia, metallic implants, or cardiac pacemakers.

### 2.2 MRI data acquisition

The same day as the neuropsychological assessments and PSG, structural and functional images were acquired with a 3.0 T GE MRI scanner (GE Discovery MR750). All subjects were instructed to lie flat with their eyes closed, stay awake, breathe steadily, and think of nothing in particular. High-resolution structural T1-weighted images were acquired for each subject with the following parameters: repetition time (TR) = 8.3 ms; echo time (TE) = 3.2 ms; field of view (FOV) = 256 × 256 mm^2^; matrix = 256 × 256; flip angle (FA) = 15°; slice thickness = 1 mm; slices = 158; scanning time = 4min27s. The rs-MRI data were obtained from each subject with an echo-planar imaging (EPI) sequence covering the whole brain based on the following parameters: TR = 2000 ms; TE = 30 ms; FOV = 224 × 224 mm^2^; matrix = 64 × 64; FA = 90°; slice thickness = 3.5 mm; slices = 33; volumes = 240; scanning time = 8 min.

### 2.3 MRI data preprocessing

MRI data analysis was performed using the software of Data Processing Assistant for Resting-State fMRI (DPARSF; Chao-Gan and Yu-Feng, 2010) with statistical parametric mapping (SPM8)^1^ and rs-fMRI data analysis toolkits (REST)^2^ based on MATLAB platform. The images were converted to the NIfTI format and the first 10 time points of rs-fMRI images were discarded because of the instability of initial MRI signals, as well as the adaptation of participants to the scanner environment. Then slice timing, head motion correction and spatial normalization to the standard Montreal Neurological Institute (MNI) space with a resampled voxel size of 3 × 3 × 3 mm^3^ were performed. The participants whose head motion >2.5 mm in translation or 2.5 in rotation were excluded in this study.

### 2.4 Calculation of ReHo and fALFF values

Before calculating ReHo values, linear detrending was performed and the preprocessed data were temporally detrended followed by regressing out nuisance covariates including Fristion’s 24-parameter regression, white matter, CSF and global mean signals. Then ReHo value, also called Kendall’s coefficient of concordance (KCC, the correlation between the time series of a given voxel and those of its nearest 26 neighbor voxels in a voxel-wise manner) was calculated to generate individual ReHo map. Finally, *Fisher’s r-to-z* transformation was performed to convert ReHo maps into zReHo (z-values) maps for normal distribution. In addition, these zReHo maps were smoothed with a 4-mm Gaussian kernel FWHM to reduce the effect of noise. The smoothed zReHo (szReHo) values were used for subsequent analysis.

Before calculating fALFF values, spatial smoothing (4-mm FWHM Gaussian kernel), detrend, and nuisance covariates regression including Fristion’s 24-parameter regression, white matter, CSF and global mean signals were performed on the preprocessed data. Then the time series of each voxel were converted to the frequency domain by the Fast Fourier transform (FFT) algorithm. The power spectrum was obtained and the square root was calculated at each frequency of the power spectrum. The average of the square root between 0.01–0.1 Hz in each voxel was calculated as ALFF value. The fALFF value was calculated as the ratio of the power spectrum of low-frequency (0.01–0.1 Hz) to that of the entire frequency range. Finally, fALFF values were standardized using *Fisher’s r-to-z* transformation (zfALFF), which could improve the normality. The zfALFF values were used for subsequent analysis.

### 2.5 Statistical analysis

Two simple *t*-tests and Chi-square test were used to assess the differences of demographic and clinical data between the groups of OSAHS and HCs using the software of Statistical Package for the Social Sciences (SPSS). The level of statistical significance was set as *p* < 0.05. Two simple *t*-tests were performed for comparing the differences of ReHo and fALFF values between the groups of OSAHS and HCs using the software of Resting-State fMRI Data Analysis Toolkit (REST) based on MATLAB platform (Song et al., 2011). The significance threshold was set at *p* < 0.001 (a minimum cluster size of 10 voxels), corrected for multiple comparisons using the AlphaSim program in REST software (Martens et al., 2018; Wu et al., 2025; Sellami et al., 2018; Park et al., 2023). Moreover, correlation analysis was performed using *Pearson* correlation analysis and the level of statistical significance was set at *p* < 0.05. Finally, ROC analysis was performed to evaluate the values of brain regions with statistically significant difference between groups for distinguishing OSAHS patients from HC, and the results for the area under the ROC curve (AUC) were displayed.

## 3 Results

### 3.1 Demographic and clinical data between OSAHS and HCs

No significant differences were identified in the age, gender, educational level and BMI between the groups between OSAHS and HCs (*p* > 0.05). OSAHS patients had decreased MoCA scores when compared with HCs (*p* < 0.01; Table 1).

**Table 1:** Demographic and clinical data between OSAHS and HCs.

Characteristics	OSAHS (*n* = 31)	HCs (*n* = 28)	*t*/χ^2^	*p*
Age (years)	42.39 ± 10.90	41.14 ± 9.16	0.47	0.64
Gender (male/female)	28/3	22/6	1.57	0.21
Educational level (years)	15.06 ± 2.99	15.68 ± 3.22	−0.76	0.45
BMI (kg/m^2^)	28.05 ± 3.53	26.75 ± 3.11	1.50	0.14
MoCA (scores)	24.39 ± 0.76	28.68 ± 1.22	−16.39	<0.01
AHI (times/h)	47.05 ± 15.06	–	–	–
SaO₂ (%)	65.26 ± 10.72	–	–	–

OSAHS, obstructive sleep apnea-hypopnea syndrome; HCs, healthy controls. BMI, body mass index; MoCA, Montreal Cognitive Assessment; AHI, Apnea Hypoventilation Index; SaO₂, Saturation of Pulse Oxygen. *p* values were obtained by two simple *t*-tests or Chi-square test. The level of statistical significance was *p* < 0.05.

### 3.2 Brain regions with altered ReHo and fALFF values in OSAHS patients

**Figure 1:**
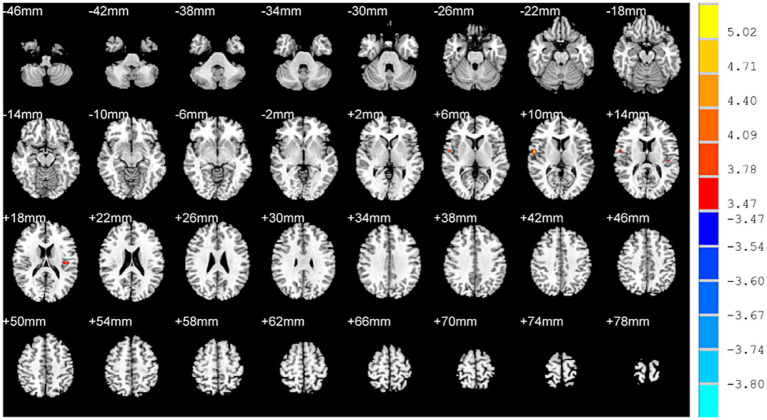
Brain regions with altered ReHo values in OSAHS patients compared with healthy controls. OSAHS: obstructive sleep apnea-hypopnea syndrome. ReHo, regional homogeneity. MIN, Montreal Institute of Neurology. The significance threshold was set at *p* < 0.001 (a minimum cluster size of 10 voxels), corrected for multiple comparisons using the AlphaSim program in REST software.

In comparison with HCs, increased ReHo values were found in the left and right rolandic operculum of OSAHS patients (Table 2; Figure 1).

**Table 2:** Brain regions with altered ReHo and fALFF values in OSAHS patients compared with healthy controls.

Brain regions	Peak MNI coordinates			Clusters	Peak *t* values
	x	y	z		
ReHo					
Left rolandic operculum	−42	−21	18	10	4.18
Right rolandic operculum	57	−3	9	25	5.33
fALFF					
Right superior frontal gyrus	18	60	18	10	−4.17
Right postcentral gyrus	30	−30	66	10	−4.65
Left angular gyrus	−45	−54	30	11	−4.54
Left thalamus	−15	−21	6	15	4.65
Right thalamus	18	−24	0	16	4.98
Right putamen	33	−12	9	28	5.13

OSAHS, obstructive sleep apnea-hypopnea syndrome. ReHo, regional homogeneity; fALFF, fractional amplitude of low-frequency fluctuation. MIN, Montreal Institute of Neurology. The significance threshold was set at *p* < 0.001 (a minimum cluster size of 10 voxels), corrected for multiple comparisons using the AlphaSim program in REST software.

**Figure 2:**
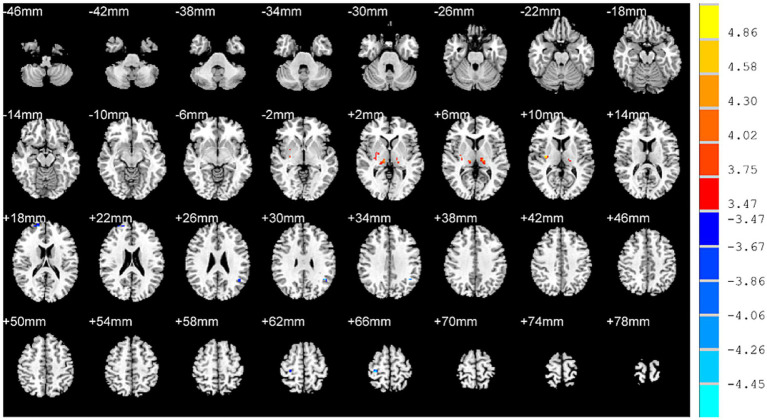
Brain regions with altered fALFF values in OSAHS patients compared with healthy controls. OSAHS, obstructive sleep apnea-hypopnea syndrome. fALFF, fractional amplitude of low-frequency fluctuation. MIN, Montreal Institute of Neurology. The significance threshold was set at *p* < 0.001 (a minimum cluster size of 10 voxels), corrected for multiple comparisons using the AlphaSim program in REST software.

In addition, decreased fALFF values were identified in the right superior frontal gyrus, right postcentral gyrus, left angular gyrus while increased fALFF values were identified in the left thalamus, right thalamus and right putamen of OSAHS patients (Table 2; Figure 2).

### 3.3 Correlations between abnormal brain regions and MoCA scores

**Figure 3:**
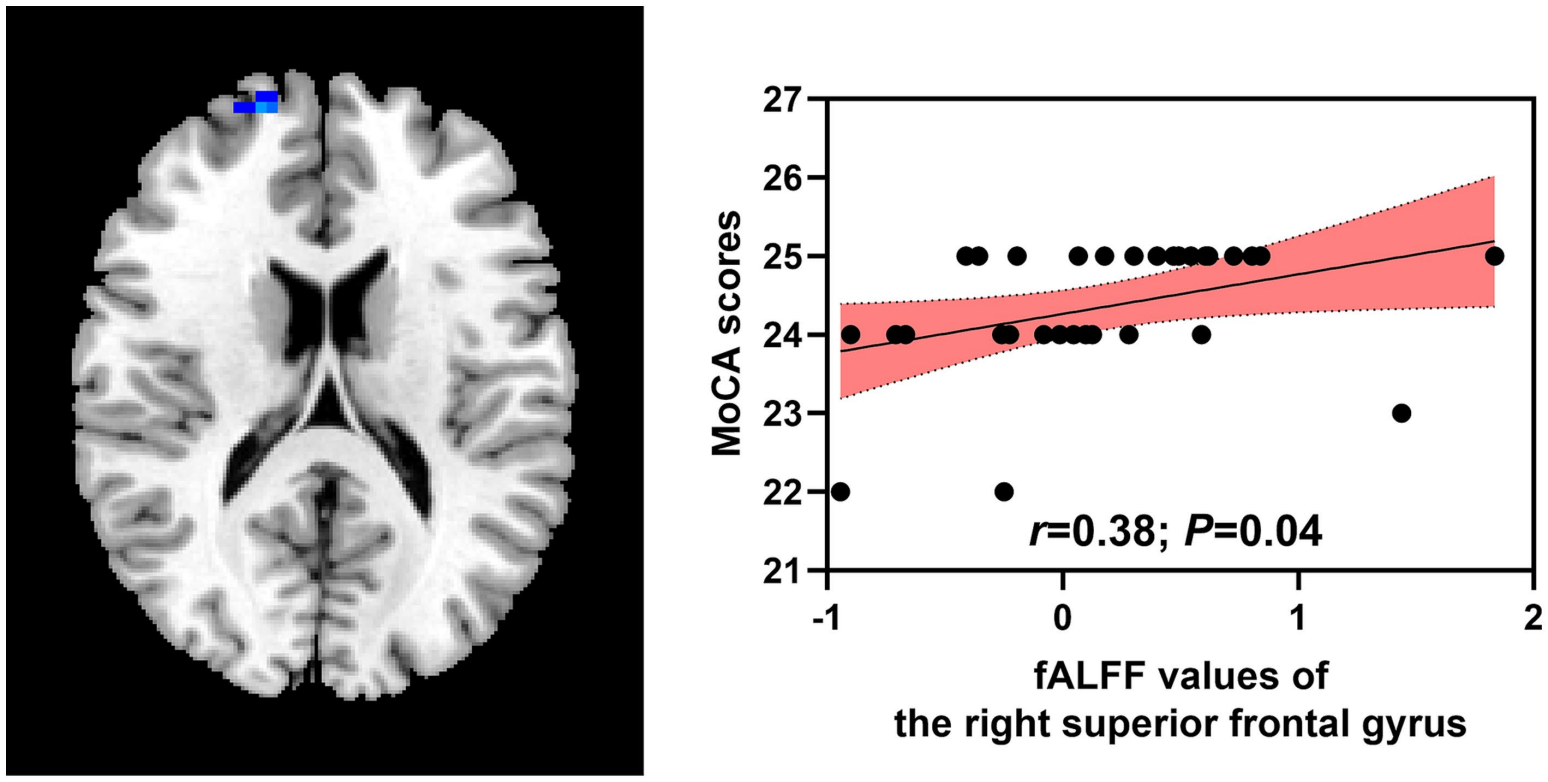
Correlations between abnormal brain regions and MoCA scores. MoCA, Montreal Cognitive Assessment; fALFF, fractional amplitude of low-frequency fluctuation.

Positive relationships were found between fALFF values of the right superior frontal gyrus and MoCA scores (*r* = 0.38; *p* = 0.04) in the patient group. No significant relationships were identified between MoCA scores and ReHo values of the left rolandic operculum (*r* = −0.22; *p* = 0.25), right rolandic operculum (*r* = −0.07; *p* = 0.72), fALFF values of the right postcentral gyrus (*r* = 0.02; *p* = 0.91), left angular gyrus (*r* = 0.26; *p* = 0.17), left thalamus (*r* = 0.25; *p* = 0.17), right thalamus (*r* = −0.70; *p* = 0.69), right putamen (*r* = −0.06; *p* = 0.75; Figure 3).

### 3.4 ROC analysis of brain regions for distinguishing OSAHS from HCs

**Figure 4:**
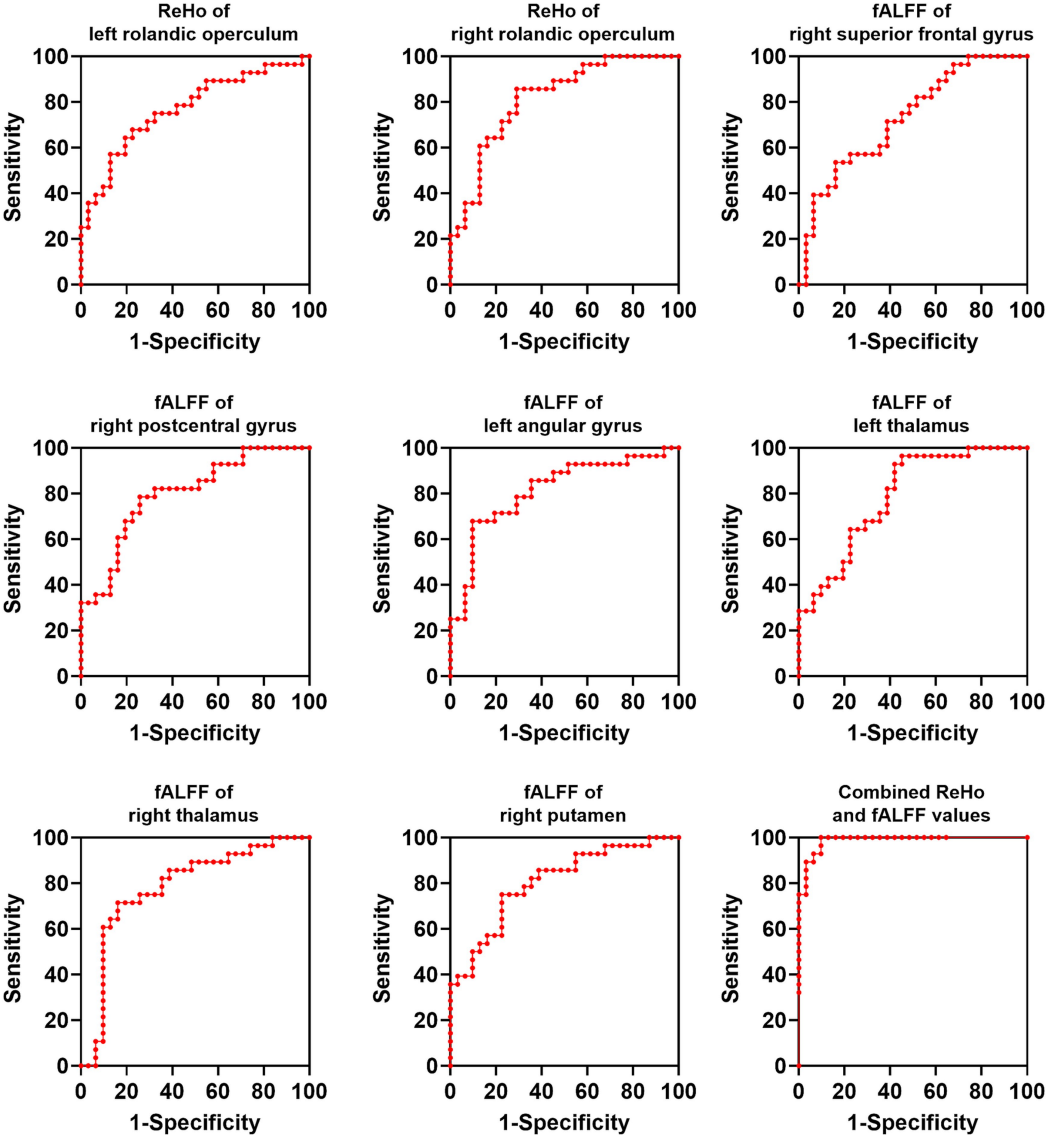
ROC analysis of brain regions for distinguishing OSAHS from HCs. OSAHS, obstructive sleep apnea-hypopnea syndrome; HCs, healthy controls. ReHo, regional homogeneity; AUC, area under the curve.

We applied a binary classification model on brain regions with altered ReHo and fALFF values to distinguish OSAHS from HCs with ReHo/fALFF values of each brain region and combined ReHo/fALFF values as test variables and OSAHS/HCs as state variables. The results of ROC analysis showed that the combined model of (ReHo and fALFF values of all abnormal brain regions) could effectively distinguish OSAHS from HCs (AUC = 0.99, sensitivity = 90.32%, specificity = 100.00%; Figure 4).

## 4 Discussion

In this study, we compared ReHo and fALFF values between severe OSAHS patients and HCs to identify brain regions with functional changes associated with OSAHS, as well as their correlations with cognitive impairment. These findings showed that severe OSAHS patients showed decreased brain activities, which were associated with the decreased cognition of patients. In addition, abnormal brain regions could help distinguishing OSAHS from HCs. These findings provided new insights about the potential pathogenesis of cognitive impairment caused by OSAHS from the perspective of changes in brain activity.

### 4.1 Changes of brain regions in OSAHS

MRI and neuropsychological testing study showed that the gray matter and metabolic levels of multiple brain regions were impaired in OSAHS patients while these patients only had mild memory and motor impairments, which might benefit from cognitive reserve and compensation mechanisms (Yaouhi et al., 2009). These findings suggested that that changes in brain structure might occur before significant neuropsychological changes in OSAHS patients. These studies emphasized the importance of early diagnosis and treatment of OSAHS. Previous study based on voxel-based morphometric (VBM) showed decreases in gray matter density in the frontal, parietal, temporal, hippocampal, and cerebellar regions of OSAHS patients (Macey et al., 2002). OSAHS patients with depressive symptoms had damages in the bilateral hippocampus and caudate nucleus, anterior part of right thalamus, and medial brainstem (Cross et al., 2008). In addition, brainstem chemosensory areas directly modulate arousal from sleep which could lead to brief arousal in OSAHS patients (Mir and Jha, 2021; Mir and Jha, 2021; Mir et al., 2019). Moreover, the depressive symptoms of OSAHS patients might exacerbate brain damage or cause additional damage to emotional, cognitive, respiratory, and autonomic control areas. Anxiety symptoms could also exacerbate brain structural damages in patients. In OSAHS patients with anxiety, structural abnormalities were identified in the anterior and middle cingulate gyrus, bilateral insular cortex, hippocampus, and temporal lobe cortex (Kumar et al., 2009).

Before treatment, OSAHS patients demonstrated decrease in gray matter density in the posterior cortex of the hippocampus and the left hippocampus (O'donoghue et al., 2005). After treatment, the memory and executive function of OSAHS patients were significantly restored, and positively correlated with increases in the gray matter volume of frontal lobe and hippocampus (Canessa et al., 2011). This neuroimaging study investigated the correlation between brain structure and cognitive function in OSAHS patients, which also evaluated the changes of brain structure after 3 months of continuous positive airway pressure treatment (Canessa et al., 2011). The results showed significant improvements in cognition, memory, executive function, and attention in patients after treatment, while cognitive and emotional impairments were closely related to the increases of gray matter volume in the hippocampus and frontal lobe, as well as the decreases of gray matter volume in the left posterior parietal cortex, left hippocampus, and right superior frontal lobe. These findings suggested that cognitive and emotional impairments of OSAHS patients could be reversed through treatment that increased the gray matter volume of specific hippocampal and frontal brain regions.

### 4.2 Mechanism of abnormal brain regions in OSAHS

OSAHS is associated with cognitive impairment and its associated brain changes are mainly located in the amygdala, hippocampus, insular, parahippocampal gyrus, and frontotemporal lobes (He et al., 2022; Lin et al., 2025; Qin et al., 2020; Chen et al., 2016). In this study, abnormal ReHo and fALFF values were identified in the brain of severe OSAHS when compared with HCs, which also showed relationships with cognitive impairment of these patients. Moreover, these changes of brain activity could help distinguishing OSAHS patients from HCs. All these findings suggested that the pathological mechanisms of cognitive impairment caused by OSAHS might be associated with changes of certain brain activity of patients.

In a previous rs-fMRI study (Chen et al., 2016), 15 Han Chinese patients with OSAHS and 15 HCs were included, the results suggested that these OSAHS patients had increased ReHo values in the right posterior lobe of cerebellum, brainstem, occipital lobe and left posterior lobe of cerebellum, basal ganglia, temporal lobe, as well as decreased ReHo values in the right middle frontal gyrus, inferior parietal lobe and bilateral superior frontal gyrus. Moreover, ReHo values of the posterior lobe of bilateral cerebellum and right middle frontal gyrus were correlated with the scores of cognitive tests. Children with OSAHS had cognitive impairments and decreased ReHo values in the right angular gyrus, precuneus and left parahippocampal gyrus, middle frontal gyrus, as well as increased ReHo values in the right posterior cerebellum (Lin et al., 2024). These patients showed increased ReHo values in the right precuneus, temporal lobe, posterior cingulate gyrus and left limbic lobe after surgery (tonsillectomy and/or adenoidectomy). These findings suggested that OSAHS children had cognitive impairment and changes in multiple brain regions, which could be improved by the treatment of tonsillectomy and/or adenoidectomy.

In China, Tibetans OSAHS patients showed increased ReHo values in the dorsolateral and medial superior frontal gyrus, as well as middle frontal gyrus (Kang et al., 2020). In addition, they had decreased ReHo values in the left fusiform gyrus and cerebellum. Moreover, increased ALFF values were found in the right orbital part of inferior frontal gyrus, middle cingulate gyrus, triangular part of inferior frontal gyrus, insula and left dorsolateral superior frontal gyrus of these OSAHS patients. However, these OSAHS patients exhibited no significant gray matter volume and functional connectivity changes when compared with Tibetans HCs, which might be due to their adaption to the hypoxia environment. In another study, OSAHS patients at high altitude showed higher ReHo values in the left superior frontal gyrus, parahippocampus and right anterior cingulate gyrus, postcentral gyrus, hippocampus, precuneus, as well as decreased ReHo values in the left cuneus and precuneus (Qin et al., 2020). These OSAHS patients also demonstrated higher ALFF values in the left medial superior frontal gyrus, parahippocampus and right anterior and middle cingulate gyrus, as well as decreased ALFF values in the left and right calcarine gyrus, right middle and inferior occipital gyrus, right cerebellum. In addition, functional connectivity between the posterior cingulate cortex and the left caudate, thalamus increased in these OSAHS patients.

### 4.3 Abnormal brain regions associated with clinical characteristics of OSAHS

In this study, positive relationships were found between fALFF values of the right superior frontal gyrus and MoCA scores in OSAHS patients. OSAHS can cause neurocognitive damage, as the degree of cognitive impairment is positively correlated with the degree of hypoxia, and is also one of the risk factors for neurodegenerative dementia (Ylä-Herttuala et al., 2021). Exploring the brain structure and corresponding functional changes involved in OSAHS can provide new insights for the pathogenesis of OSAHS and its reversibility in this disease. With the development of MRI technology, multiple imaging studies have found that OSAHS patients have changes of activity and functional connections in certain brain regions, which provide a deeper understanding of OSAHS (Qin et al., 2020; Chen et al., 2016; Lin et al., 2024; Kang et al., 2020). These changes provide important insights into the pathological mechanisms of OSAHS and help researchers recognize that certain changes may be associated with the clinical symptoms, decline in cognitive function, and emotional changes of patients. The ReHo values of bilateral inferior parietal lobes, right superior temporal gyrus, and left precentral gyrus were reduced in patients with moderate to severe OSAHS (Li et al., 2021). One month of CPAP treatment could partially reverse the spontaneous activity of abnormal brain regions and produce adaptive changes in OSAHS, such as bilateral posterior cerebellar lobes, right superior temporal, and left precentral gyrus. The changes in MoCA of patients before and after treatment were significantly correlated with the average ReHo values of the right inferior parietal lobule. This study provided neuroimaging evidences, revealing that therapy could have certain effects on the structural changes of the brain in OSAHS patients.

### 4.4 Advantages of using rs-fMRI in OSAHS

Based on the findings of this study, the additional value of rs-fMRI assessing cognitive level of OSAHS patients in the clinical practice include (1) repeatability: the rs-fMRI examination process is relatively simple and not limited by the patient’s physical condition, allowing for multiple examinations to observe the dynamic changes in cognitive dysfunction in OSAHS patients, providing a basis for evaluating treatment effectiveness and predicting disease prognosis; (2) multi parameter analysis: rs-fMRI can evaluate the functional status of the brain through various parameters and analysis methods, which can reflect the functional activity of the brain from different perspectives, providing more comprehensive information for the assessment of cognitive impairment in OSAHS patients; (3) early diagnostic potential: rs-fMRI can detect early changes in brain function in OSAHS patients before significant cognitive impairment symptoms appear, which is helpful for early diagnosis and intervention. For OSAHS patients, early detection and intervention of cognitive impairment can delay the progression of the disease and improve the patient’s quality of life.

### 4.5 Limitations of this study

However, several limitations should be addressed in this study. This a cross-sectional study and the sample size the present study was relatively small. In addition, we did not include rs-fMRI data of patients after the treatment. Therefore, longitudinal studies with relatively larger sample sizes were needed to elucidate the mechanisms of treatment on the brain activity of OSAHS patients based on the longitudinal follow-up rs-fMRI data. Moreover, multimodal MRI brain imaging studies (structural MRI, diffusion MRI and functional MRI) were required to comprehensively evaluate both structural and functional changes in the brain of OSAHS patients. Finally, external validation was not performed in this study for distinguishing OSAHS patients from HCs. Therefore, additional MRI data should be collected in further studies and the external validation should be conducted to validate these findings. Future studies using task-based fMRI paradigms would be conducted to enhance diagnostic reliability and contribute to developing more predictive models of cognitive dysfunction in OSAHS.

## 5 Conclusion

In this study, both ReHo and fALFF values were utilized to investigate the brain functional changes of severe OSAHS when compared with HCs, as well as their relationships with cognitive impairment. The results showed that these OSAHS patients showed decreased brain activities, which were associated with the decreased cognition of patients. In addition, abnormal brain regions could help distinguishing OSAHS patients from HCs. These findings provided new insights about the pathological mechanisms of cognitive impairment caused by OSAHS and help recognizing that changes of certain brain activity might be associated with the clinical symptoms of patients.

## Data Availability

The raw data supporting the conclusions of this article will be made available by the authors, without undue reservation.

## Footnotes

^1^ http://www.fil.ion.ucl.ac.uk/spm/

^2^ http://www.restfmri.net/
